# Seasonal changes in photoperiod and temperature lead to changes in cuticular hydrocarbon profiles and affect mating success in *Drosophila suzukii*

**DOI:** 10.1038/s41598-023-32652-y

**Published:** 2023-04-06

**Authors:** Zsolt Kárpáti, Ferenc Deutsch, Balázs Kiss, Thomas Schmitt

**Affiliations:** 1 Department of Animal Ecology and Tropical Biology, Biocenter, University of Würzburg, Würzburg, Germany; 2 Zoology Department, Plant Protection Institute, Centre of Agricultural Research, ELKH, Budapest, Hungary; 3 Hungarian University of Agriculture and Life Sciences, Gödöllő, Hungary

**Keywords:** Ecology, Zoology

## Abstract

Seasonal plasticity in insects is often triggered by temperature and photoperiod changes. When climatic conditions become sub-optimal, insects might undergo reproductive diapause, a form of seasonal plasticity delaying the development of reproductive organs and activities. During the reproductive diapause, the cuticular hydrocarbon (CHC) profile, which covers the insect body surface, might also change to protect insects from desiccation and cold temperature. However, CHCs are often important cues and signals for mate recognition and changes in CHC composition might affect mate recognition. In the present study, we investigated the CHC profile composition and the mating success of *Drosophila suzukii* in 1- and 5-day-old males and females of summer and winter morphs. CHC compositions differed with age and morphs. However, no significant differences were found between the sexes of the same age and morph. The results of the behavioral assays show that summer morph pairs start to mate earlier in their life, have a shorter mating duration, and have more offspring compared to winter morph pairs. We hypothesize that CHC profiles of winter morphs are adapted to survive winter conditions, potentially at the cost of reduced mate recognition cues.

## Introduction

Seasonal polymorphism and phenotypic plasticity have been documented in several insect species. Phenotypic plasticity is brought by altered gene expression as a consequence of adaptation to different environmental conditions. For instance, the butterfly *Bicyclus anynana* exhibits wing pattern plasticity in response to wet and dry seasons^1^. Another well-known example of polymorphism is the winged and wingless aphid morphs that develop in response to different environmental conditions such as crowding, interspecific interaction and abiotic factors (reviewed in^2^). One of the most common forms of seasonal plasticity in insects is diapause, a reversible state of developmental arrest and metabolic restructuring that insects enter when exposed to harsh conditions, such as cold climate^2–6^. Many insects undergo adult reproductive diapause, which is a specific type of plasticity characterized by the arrested development of reproductive organs and suspended reproductive activity in response to unfavorable environmental conditions^3,7^. Reproductive diapause occurs in several *Drosophila* species, including *D. melanogaster*, however, it can vary among geographically dispersed populations^7–10^.

Cuticular hydrocarbons (CHCs), which cover the body surface of insects, play an important role in protecting them against desiccation and, therefore, help them survive cold climatic conditions^11^. CHCs are also essential in chemical communication and may signal species identity, nestmate identity, sex and mating status^11–13^. The CHC profile is a complex mixture of hydrocarbons with diverse chain lengths including *n*-alkanes, methyl-branched alkanes, and unsaturated hydrocarbons^11^. In the genus *Drosophila*, CHCs have functions as aggregation pheromones and for mate and species recognition in addition to protection against desiccation. For example, *D. melanogaster* males produce and deposit 9-tricosene as an aggregation pheromone on valuable food sources to attract females^14^. Although CHC profiles are often species and sex-specific they might be affected by changing environmental factors such as temperature, humidity, or day length^15,16^.

The plasticity of CHC compositions over a lifetime according to different environmental conditions has been reported for several insect species^11,17–19^. In *D. melanogaster,* individuals respond to drought stress by increasing the biosynthesis of alkanes, which protects them against desiccation better than methyl-branched or unsaturated hydrocarbons^20,21^. Cold-adapted *Drosophila montana* shifts its CHC profile toward hydrocarbons with higher chain-length during reproductive diapause in winter, while simultaneously losing shorter chain-length hydrocarbons suggested cues for sexual attraction and communication^22^.

In this study, we focus on the invasive spotted wing drosophila, *Drosophila suzukii* (Matsumura) (Diptera: Drosophilidae), which is one of the greatest threats to fruit production in temperate zones worldwide including the US and Europe^23–25^. This species is polyphagous and oviposits in all soft skin fruits which permits penetration by its morphologically adapted serrated ovipositor^23,26^. Although recognition of host plants and host finding is heavily studied in *D. suzukii*^27,28^, we have only scarce knowledge about the intraspecific chemical communication of this species. Dekker et al.^29^ reported that *D. suzukii* males do not produce cVA (cis-11-octadecenyl acetate), which is a generally used pheromone component in the melanogaster group, and the CHC profiles of *D.suzukii* are not sexually dimorphic. Another study demonstrates that 3-day-old *D. suzukii* females produce quantitatively more CHCs than 2-day-old females. This increase in CHC production was associated with increased mating activity^30^. Furthermore, Snellings et al.^31^ confirmed that this species has a sexually monomorphic CHC profile. In addition, they identified three CHCs, namely 9-tricosene, 7-tricosene and tricosane, which increased in relative abundance with age and maturity. Interestingly, a significant reduction in mating success was observed when these three compounds were artificially applied to the female abdomen.

*Drosophila suzukii* exhibits seasonal phenotypic plasticity that causes reproductive diapause, leading to morphologically different summer and winter morphs^32^. The winter morph has longer wings and stronger abdominal melanization^33^. It develops from pupae experiencing lower temperatures and photoperiods with shorter daylight^32,33^. The winter morph survives better at low temperatures compared to the summer morph, supporting the observation that this species can persist in areas with cold winters^32,33^. The down-regulation of gene transcripts involved in oogenesis and DNA replication in winter morph supports reproductive diapause^33^. However, CHC profiles of the two morphs of *D. suzukii* have not been comparatively studied; although, it might help to understand the better survival rate under winter conditions and reproductive diapause traits.

In this study, we hypothesize that CHC profiles of *D. suzukii* will differ between summer and winter morphs, which will facilitate the flies’ survival in winter. Specifically, we expect that the winter morphs would have a higher proportion of long-chain hydrocarbons, which have been shown in previous studies to be important for cold tolerance. We also hypothesize that the altered CHC profile may affect the flies’ mating, via the reduction or elimination of the necessary chemical cues for mate recognition. To test these hypotheses, we identified and compared CHC profiles of 1- and 5-day-old, males and females of summer and winter morphs. In addition, we examined the mating success and the number of offspring of all combinations of both morphs within the same age group.

## Results

### Characterization and comparison of cuticular hydrocarbons

Overall, we identified CHCs ranging in chain length from C17 to C33 including alkanes, monomethyl-branched and dimethyl-branched alkanes, as well as alkenes and alkadienes (Table S1). We quantified 77 compounds from all groups, 69 of which we were able to identify (Table S1). Across all groups, we found only one dimethyl-branched alkane (7,17-dimethylnonacosane), occurring in 6 out of 8 experimental groups (Table S1). We identified 55, 52, 57 and 64 CHCs in 1-day-old summer morph females and males, and winter morph females and males, respectively. 5-day-old summer morph females and males produce fewer CHCs: 32 and 28, respectively. However, 5-day-old winter morphs kept under summer conditions for five days produce almost as many CHCs (50 in males and 43 in females) as 1-day-olds (Table S1).

**Figure 1:**
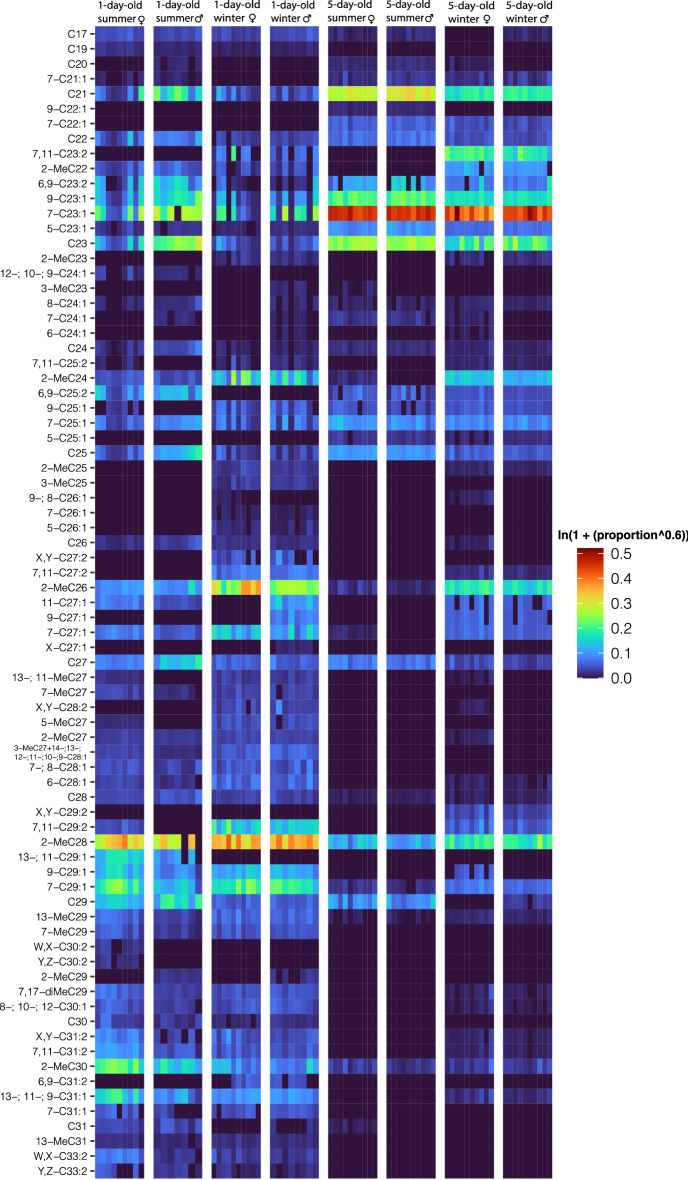
Heat map of relative abundance of 77 identified CHCs from *D. suzukii.* W, Z, X, Y—double bond position was not identified. Data were transformed ln(1 + (proportion^0.6)) and are displayed in colors ranging from dark blue (indicates 0) to dark red (indicates 0.5) as shown in the key.

To visualize the differences between winter and summer morphs, sexes and age groups, we produced a heat map based on the relative amounts of each hydrocarbon (Fig. 1). The heat map shows that 1-day-old summer morph females and males produce more shorter-chain CHCs from a chain length of C19 to C24 compared with same age winter morphs. The 5-day-old females and males show an even more pronounced shift to shorter-chain hydrocarbons of the same chain length. Thus, only 1-day-old winter morphs who are in reproductive diapause produce a low number of shorter-chain CHCs. The heatmap also shows that all 5-day-old individuals have less longer-chain CHCs from C27 to C33 compared with 1-day-old individuals (Fig. 1).

**Figure 2:**
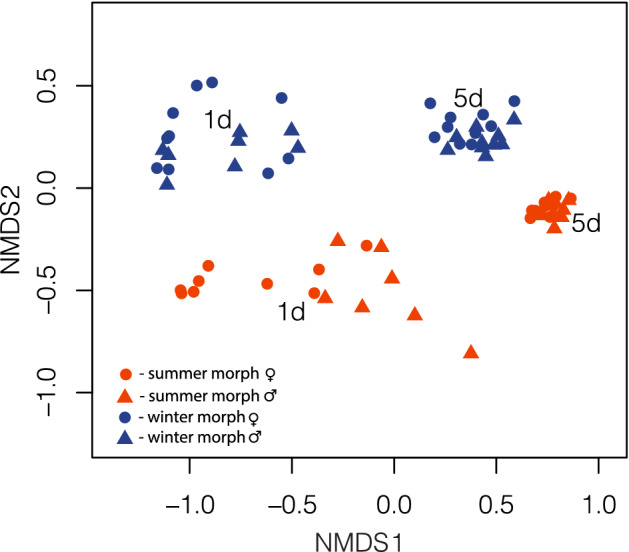
Nonmetric multidimensional scaling (NMDS) based on Bray–Curtis distance between samples. Data is displayed in a two-dimensional graph (stress = 0.053).1d: 1-day-old, 5d: 5-day-old.

Finally, we found significant CHC profile differences between winter and summer morphs (F_1,72_ = 17.484; *p* = 0.001; Fig. 2) and between 1- and 5-day-old adults (F_1,72_ = 107.57; *p* = 0.001; Fig. 2). However, there was no significant difference between the CHC profiles of males and females (F_1,72_ = 2.202; *p* = 0.1; Fig. 2).

### Mating trials

**Figure 3:**
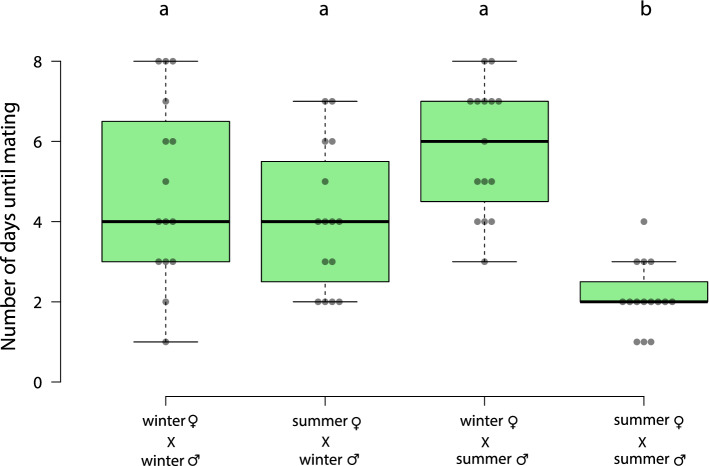
Box plot representing days until the mating event for *D. suzukii*. Center lines show the medians; box limits indicate the 25th and 75th percentiles, whiskers extend 1.5 times the interquartile range from the 25th and 75th percentiles, n = 15 mating pairs per treatment. All data were analyzed by the Kruskal–Wallis test (H = 25.7; *p* < 0.0001 with post hoc analysis (Dunn’s test); different letters indicate significant differences (*p* < 0.05).

We conducted behavioral assays to evaluate the mating success of inter- and intramorph pairs of *D.suzukii*. We examined the number of days since eclosion required for mating to begin, the duration of mating and the number of adult offspring. Summer morph males and females begin mating significantly earlier than pairs with either males or females of the winter morph or both winter morphs (Fig. 3; Dunn’s test: summer♀ X summer♂ vs. winter♀ X winter♂ *p* = 0.002; summer♀ X summer♂ vs. summer♀ X winter♂ *p* = 0.041; summer♀ X summer♂ vs. winter♀ X summer♂ *p* < 0.001). There is no significant difference in the number of days needed for mating between pairs that have winter morphs as one or both of their members (Fig. 3).

**Figure 4:**
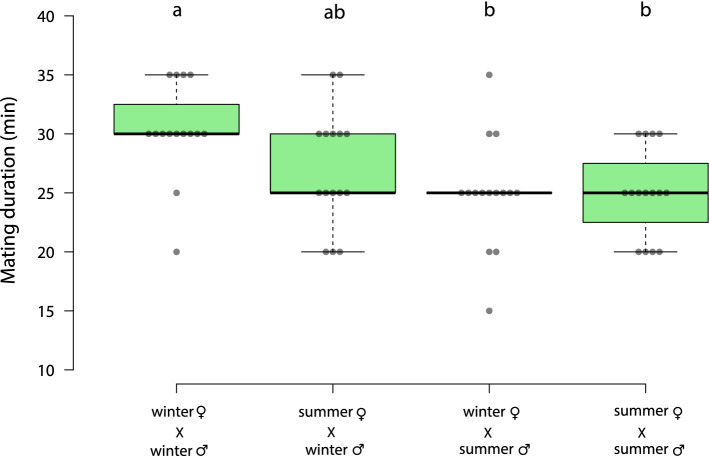
Box plot representing the mating duration of *D. suzukii*. Center lines show the medians; box limits indicate the 25th and 75th percentiles, whiskers extend 1.5 times the interquartile range from the 25th and 75th percentiles, n = 15 mating pairs per treatment. One-way ANOVA (effect of mating group F = 5.00 *p* < 0.004) with Tukey’s multiple comparisons post hoc test was performed. Different letters show significant differences (*p* < 0.05).

The duration of mating significantly differed between the four types of mating pairs (one-way ANOVA, F(3, 56) = 5.00, *p* = 0.038). The mating was significantly longer when both sexes were winter morphs, compared with either winter morph females mated with summer morph males or summer morph females mated with summer morph males (Fig. 4; winter♀ X winter♂ vs. winter♀ X summer♂ *p* = 0.008; winter♀ X winter♂ vs. summer♀ X summer♂ *p* = 0.008). There was no significant difference in mating duration between winter morph females mated with winter morph males and summer morph females mated with winter morph males (Fig. 4; winter♀ X winter♂ vs. summer♀ X winter♂ *p* = 0.17).

**Figure 5:**
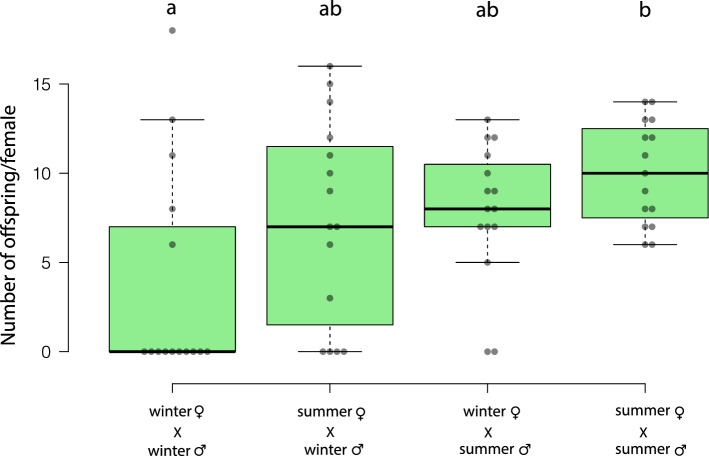
Box plot representing the offspring per female of *D. suzukii*. Center lines show the medians; box limits indicate the 25th and 75th percentiles, whiskers extend 1.5 times the interquartile range from the 25th and 75th percentiles, n = 15 mating pairs per treatment. All data were analyzed by the Kruskal–Wallis test (H = 10.1; *p* = 0.02) with post hoc analysis (Dunn’s test); different letters indicate significant differences (*p* < 0.05).

After the mating observations, we counted the adult offspring of the mating pairs. The numbers of eclosed offspring of the intra- and intermorph pairs differ significantly between the groups (Fig. 5; Kruskal–Wallis test, χ^2^ = 9.85, n = 60, *p* = 0.012). There is a significant difference between offspring of intramorph pairs, i.e. summer morph females mated with summer morph males produced more offspring than winter morph females mated with winter morph males (Fig. 5; Dunn’s test: winter♀ X winter♂ vs. summer♀ X summer♂ *p* = 0.01). All other pairs did not show any significant differences (Fig. 5; Dunn’s test: winter♀ X winter♂ vs. summer♀ X winter♂ *p* = 0.42; winter♀ X winter♂ vs. winter♀ X summer♂ *p* = 0.27; summer♀ X summer♂ vs. summer♀ X winter♂ *p* = 1; summer♀ X summer♂ vs. winter♀ X summer♂ *p* = 1).

Taken together, these results revealed that summer morph pairs mated earlier as adults mated for a shorter time period and had more adult offspring than winter morph pairs.

## Discussion

Reproductive diapause is a crucial strategy for many insect species in temperate zones to survive the winter^6^ and, thus, occurs also in most temperate *Drosophila* species^34,35^ including *D. suzukii*^33^. The latter species shows a seasonal dimorphism as an adaptation to different climatic conditions in summer and winter. The winter morph develops different morphological traits as longer wings and stronger abdominal melanization compared to the summer morph^33^. To understand whether CHCs as a physiological trait are affected during the development of the different morphs, we identified and compared the CHC profiles of the two morphs at two different ages. In addition, we performed behavioral tests on intra- and intermorphic pairs to determine whether the CHC profile changes correlate with changes in mating success.

*Drosophila suzukii* shows an age-dependent CHC profile developing from 1-day-old to 5-day-old individuals by reducing the number and relative amounts of longer-chain hydrocarbons in favor of shorter-chain hydrocarbons. There is evidence that CHC profiles containing more longer-chain hydrocarbons have evolved in several species of *Drosophila* against desiccation stress^36^. However, not all species-specific CHC profile compositions can be explained by adaptation to environments with low humidity or high temperatures^37,38^. Both, shorter-chain and longer-chain hydrocarbons can function as sex pheromones in *Drosophila* as shown in *D. melanogaster*. A male-specific aggregation pheromone in *D. melanogaster* is 7-tricosene, whereas female sex pheromones are identified as two alkadienes with the chain-length of C27 and C29^39^. Age-dependent, quantitative differences in CHC production comparing summer morphs of sexually immature and mature *D. suzukii* males and females have already been described^31^. Similarly, Revadi^30^ reported quantitative CHC differences with age of *D. suzukii* females correlating with higher mating activity in older individuals. Age-dependent changes in CHCs are also known from other *Drosophila* species. For instance, it has been shown that the composition of CHCs in *D. melanogaster* males and females changes with age^40^. Also, the total amount of CHCs produced by *D. virilis* increases in an age-dependent manner; moreover, this species also exhibits qualitative differences by producing more alkenes with age^41^. In these studies, however, the function of the age-related CHC changes has not been evaluated. We hypothesize that these age-dependent CHC profile differences in *D. suzukii* may be involved in courtship and short-range mate recognition. We could also confirm that summer morph *D. suzukii* does not produce sexually dimorphic CHC profiles in both 1- and 5-day-old individuals^29,31^.

According to our knowledge, the CHC profile of the winter morph *D. suzukii* has not yet been characterized. We found significant differences in CHC composition between summer and winter morphs, whereby winter morphs produced more long-chain methyl-branched and unsaturated hydrocarbons in 5-day-old individuals. In 1-day-old individuals, the winter morphs show less amounts of short-chain hydrocarbons (C21 to C23) compared to the summer morph of the same age. In addition, also CHC profiles of winter morphs did not show sexual dimorphism. Interestingly, a seasonal difference has also been shown in diapausing *D. montana.* Their CHC profile differed from reproductively active ones by shifting from short-chain to long-chain CHCs^22^.

We suggest that the CHC profile differences between summer and winter morphs are an adaptation to different climatic conditions which has consequences for the mating success of these morphs. We found that if 1-day-old pairs included at least one fly of the winter morph, their latency of mating will be on average 4–6 days. In contrast, 1-day-old summer morph males and females mate within two days. These differences in behavior might be explained by the development of CHCs of 1-day-old winter males and females from a non-attractive to an attractive profile as we already know that CHCs play an important role in mate recognition in *D. suzukii*^31^. Alternatively, the latency of mating could be explained by the immaturity of the individuals of the 1-day-old winter morph. To test these hypotheses, perfuming experiments should be conducted as shown in Davis et al.^37^. Ala-Honkola et al.^22^ reported similar CHC profile differences between diapausing and reproductive adults of *D. montana*, where mating success correlated with CHC profiles, i.e., diapause-induced pairs had lower mating rates and offspring, corresponding to the differences in CHC profiles of diapausing and non-diapausing individuals. Interestingly, there was also no sexual dimorphism in the CHC profiles in *D. montana* leading to courtship behavior of males towards females and males. This courtship behavior was only performed by males towards non-diapausing individuals hinting towards a function of short-chain CHCs as recognition cues. In *D. suzukii* there are also no sexually dimorphic CHC profiles. In congruence with the findings in *D. montana*, seasonal changes in composition of their CHC profile may still affect mate recognition. Moreover, our behavioral assays also show that mating duration of winter morph pairs was significantly longer than compared with summer morph pairs. Elongated mating duration is often linked to extended mate guarding^42^ or to ensure ejaculate transfer^43,44^. If both sexes are winter morphs in *D. suzukii*, it seems more plausible that immature individuals increase the mating duration to optimize the number of offspring instead of males mate-guarding females. Interestingly, elongated mating did not result in a higher number of offspring. In contrast, winter morph pairs have significantly fewer offspring than summer morph pairs. The decrease in mating success in the winter morph might indicate the investment into the survival of the cold season. It is consistent with the results of the field-collected *D. suzukii*, where winter morph females produce fewer eggs than summer morph females^45–47^. The low number of winter morph offspring may partly explain the population dynamics with a slow increase in population size in spring to a massive increase in the summer and autumn^48–50^.

CHC differences between the two morphs may derive from the need of the winter morph to adapt to winter conditions by synthesizing a CHC profile which protects from cold temperatures and desiccation stress. According to Panel et al.^51^, during 31 days of cold exposure, both sexes of the winter morph of *D. suzukii* have significantly higher survival rates compared to the summer morph. Furthermore, Wallingford et al.^46^ showed that the winter morph survived more successfully at − 1 °C for 24 h than the summer morph. In addition, *D. suzukii* winter and summer morphs exhibit differences in gene expression, which may contribute to the biosynthesis of the different CHC profiles^33^. In winter, having an altered CHC profile offers an evolutionary advantage of surviving cold climates, however, the disadvantage might be the potential for weak or no sexual signals. The CHC profile of *D. montana*, for example, differed from reproductively active ones by shifting from short-chain to long-chain CHCs in diapausing phenotypes. The authors of the study hypothesized that males and females were less attracted to each other due to the absence of short-chain CHCs which may be used as sex pheromones^22^, similar to what we find in *D. suzukii*. However, experimental evidence of the absence of sex pheromones is not provided, either in the study of *D. montana*, or in the present study of *D. suzukii*. Alternatively, immaturity of the 1-day-old *D. suzukii* males and females may explain the latency in mating. In face flies (*Musca autumnalis*) CHC composition also drastically changes during diapause, producing a higher amount of methyl-branched alkanes and a lower amount of alkenes suspending mating behavior^15^.

Finally, we hypothesize that the shift of the CHC profiles of the *D. suzukii* winter morph to adapt to cold climatic conditions. However, mate recognition cues are restored in the winter morph within five days if they are exposed to summer conditions. The CHC changes from diapause to non-diapause profiles are similar to those found in *D. montana*^22^ and they may also occur in other diapausing *Drosophila* species.

## Materials and methods

### Insects

The *D. suzukii* rearing was established in 2019 with approximately 250 individuals emerging from cultivated blackberries (*Rubus fruticosus* ‘Loch Ness’) in Hungary, near Romhány (47° 54′ 49.2″ N 19° 14′ 57.1″ E). Flies were reared on a standard alfalfa/wheat germ diet^52^ at 23 °C, 60% RH under 16L:8D. To produce imagines for the experiments, final stage larvae leaving the medium for pupation were isolated in individual glass vials (0.5 cm ID, 6 cm length) and were randomly assigned to two groups for producing different morphotypes. To obtain summer morphs, the pupae were kept at 23 °C, 60% RH and 16L:8D. To obtain winter morphs, the pupae were kept at 12 °C, 60% RH and 12L:12D. The morphotype of all imagines were verified under a stereo microscope based on their melanization of the abdominal tergites as described in Panel et al.^49^. After emerging, flies of both groups were kept at their rearing conditions for 24 h. Both groups were kept under summer conditions after the first 24 h.

### Analysis of cuticular hydrocarbons

We analyzed the CHC profiles of 1- and 5-day-old males and females of summer and winter morphs of *D. suzukii* (1-day-old summer morph males: n = 7, 1-day-old winter morph males: n = 8, 1-day-old summer morph females: n = 9, 1-day-old winter morph females: n = 10, 5-day-old summer morph males: n = 10, 5-day-old winter morph males: n = 10, 5-day-old summer morph females: n = 10 and 5-day-old winter morph females: n = 10). Individual flies were killed at − 20 °C. Thawed individuals were immersed in 40 μL *n*-pentane (SupraSolv, Merk, Darmstadt, Germany) for 5 min using glass inserts placed in chromatography vials. 10 μL of 1 ng/μL octadecane (purity: 99.5%, Fluka, Buchs, Switzerland) in *n*-heptane was added to the extracts as an internal standard. Each extract was transferred to another glass insert and concentrated under a gentle flow of CO_2_ to 4–5 μL. Using auto-injection, the total extract was injected into a gas chromatograph coupled to a mass selective detector (GC–MS) (Agilent 7890B GC and 5977 MS, Agilent Technologies, Waldbronn, Germany) using a split/splitless injector in splitless mode at 300 °C. The GC was equipped with an HP-5MS UI capillary column (30 m × 0.25 mm × 0.25 μm, J and W Scientific, Folsom, CA, USA). Helium was used as carrier gas with a flow rate of 1 mL min^−1^. The initial GC oven temperature was 60 °C held for 1 min, then raised to 300 °C at 5 °C min^−1^ where it was held for 10 min. The transfer line temperature between GC and MS was 300 °C. The mass spectrometer was operated in electron impact (EI; 70 eV) ionization mode, scanning m/z from 40 to 650, at 2,4 scans s^−1^.

Cuticular hydrocarbons were identified based on their characteristic ions. The identification was verified by comparison with the calculated retention indices (RIs)^53^. We used dimethyl disulfide (DMDS) derivatization to locate the double bold positions in alkenes and alkadienes^54^. Integrated peak areas were used to calculate the relative amount of each compound. Our internal standard was used to calculate each compound's absolute amount. Compounds were further analyzed if they are present in at least 50% of the samples of a given group and contribute more than 0.1% of the total CHC abundance. In the case of the non-accurately separated compounds, the combined peak area and quantity have been calculated.

We created a heatmap (R software, version 4.2.2, package ggplot2^55^) to visualize the relative differences of CHC compounds between morphs, sexes and ages. The data for the heatmap was calculated with the natural logarithm of (1 + (proportion^0.6)) for each compound to display color differences of different proportions of substances.

To visualize the chemical distances, we used non-metric multidimensional scaling (NMDS), based on a Bray Curtis dissimilarity matrix. In the NMDS plot, the spatial distances between points indicate the differences in CHC profiles between samples, while the stress value indicates how well the two-dimensional representation fits the multidimensional distances^56,57^. Data structures derived from the visualization method do not require a priori knowledge of samples representing a group. Grouping is solely based on the chemical compositions of the analyzed extracts. PERMANOVA (Permutational multivariate analysis of variance, permutations = 9999) was applied to test for differences between winter and summer morphs, sexes and ages^58^. The statistical analyses were performed in R^59^ using the vegan package^60^.

### Mating trials

Four groups of males and females were generated for the behavioral assays: (1) summer morph female with summer morph male (n = 15), (2) summer morph female with winter morph male (n = 15), (3) winter morph female with winter morph male (n = 15) and (4) winter morph female with summer morph male (n = 15) (see also in Table S2). Each mating trial was started 24 h after the adult flies emerged. Flies were placed individually in glass vials (2.5 cm ID, 4.5 cm length) and fed with sucrose solution. On the first day of the mating trials, all flies were placed under summer conditions one hour before the mating trial started to ensure a convenient temperature for mating behavior. At the beginning of the trials, individual males were transferred to the females’ vials. Pairs were allowed to start mating for 1 h each consecutive day between 11:00 and 14:00. We registered the time of the beginning and the end of mating. After the observation, males were removed from the females’ vials. If mating did not begin within an hour, males were placed again into the corresponding female's vial each consecutive day until successful mating. All pairs successfully mated within 8 days. If one of the mating partners died before mating the pair was discarded from the experiment (Table S2). If a successful mating was observed, the female was placed in another glass vial (2 cm ID, 9 cm length) containing the same medium as mentioned above for feeding and oviposition. The vials were kept in a climate chamber (23 °C L:D 16:8) until the females died and all the adult offspring emerged. The number of offspring (number of individuals reaching the adult stage) for each vial was counted.

Kruskall-Wallis rank sum test was used to test differences in mating delay and the number of progenies between groups since the data were not normally distributed (verified with the Kolmogorov–Smirnov test). We conducted Dunn’s post-hoc test with Bonferroni *p*-value correction to determine differences between groups. Differences in mating duration between groups were tested with a one-way ANOVA and Tukey HSD post-hoc test using Past 4.03 software^61^.

### Ethics declarations

The invertebrate insect species (spotted wing Drosophila, *Drosophila suzukii*) used in the present study has a horticultural pest status and is not protected in Europe, including Hungary. Therefore, individuals can be freely collected and used in laboratory experiments without a permit or approval from the institutional ethics committee or national authorities under Hungarian law (348/2006, paragraph 10/3).

The owner of the blackberry field, Attila Kakukk (self-employed, Berkenye, Hungary) provided full permission to collect blackberries (*Rubus fruticosus*, 'Loch Ness' variety), which were infested with *D. suzukii*. The collection of blackberries (*Rubus fruticosus*, 'Loch Ness' variety) has complied with relevant institutional, national, and international guidelines and legislation. The blackberry (*Rubus fruticosus*, 'Loch Ness' variety) is a commercially available, commonly cultivated plant. This plant species is neither protected nor endangered.

## Supplementary Information

Supplementary Information.

## Data Availability

All data are presented in the manuscript and in the supplemental material.
