# Supplementary material for: Seasonal changes in photoperiod and temperature lead to changes in cuticular hydrocarbon profiles and affect mating success in Drosophila suzukii
Source: Sci Rep. 2023 Apr 6;13:5649. doi: 10.1038/s41598-023-32652-y (PMC10079849; doi:10.1038/s41598-023-32652-y)
Supplement: Supplementary file 1 — Supplementary Information. [file 41598_2023_32652_MOESM1_ESM.pdf]

Seasonal changes in photoperiod and temperature lead to changes in cuticular hydrocarbon profiles and affect mating success in *Drosophila suzukii*

Zsolt Kárpáti [1,2*], Ferenc Deutsch[2,3], Balázs Kiss[2], Thomas Schmitt[1]

[1] Department of Animal Ecology and Tropical Biology, Biocenter, University of Würzburg, Würzburg, Germany
[2] Zoology Department, Plant Protection Institute, Centre of Agricultural Research, ELKH, Budapest, Hungary
[3] Hungarian University of Agriculture and Life Sciences, Gödöllő, Hungary

[*] corresponding author

Supplementary material

Supplementary Table S1: List of cuticular hydrocarbons identified in *D. suzukii* extracts with calculated mean amount in ng per fly and standard errors. RI = Retention index. W, Z, X,Y - double bond position was not identified.

Compound name	RI	1-day-old-summer♀	1-day-old-summer ♂	1-day-old-winter♀	1-day-old-winter ♂	5-day-old-summer♀	5-day-old-summer ♂	5-day-old-winter♀	5-day-old-winter ♂
C17	1700	8.22±1.12	5.3±0.54	5.55±0.81	5.25±0.53	2.06±0.28	2.45±0.31		1.08±0.36
C19	1900	2.67±0.39	1.11±0.13	0.81±0.1	0.73±0.08				
C20	2000		1.16±0.36			3.12±0.54	2.98±0.41	0.85±0.24	
7-C21:1	2079	0.99±0.4	1.07±0.44			5.48±1.89	5.73±0.92	5.53±3.26	4.72±2.07
C21	2100	30.59±19.07	89.46±27.94	14.82±5.68	8.49±2.47	505.35±85.46	377.01±50.18	131.84±36.69	91.26±15.35
9-C22:1	2172					2.83±0.35	2.18±0.32		
7-C22:1	2179					25.73±3.45	18.88±2.55	12.26±4.44	6.9±1.88
C22	2200	19.19±7.73	19.91±3.35	4.64±1.2	4.59±0.85	34.62±3.87	28.22±3.84	10.86±2.96	6.95±2.11
7,11-C23:2	2260			26.47±15.27	9.3±2.89			177.76±61.85	74.89±11.52
2-MeC22	2263	10.65±3.57	12.19±3.94	6.11±2.04	6.99±2.23			35.48±10.32	23.24±4.35
6,9-C23:2	2269	33.43±14.13	53.26±15.79	4.63±2.78	6.4±5.13	78.38±15.41	67.41±25.37	52.12±36.48	31.89±16.3
9-C23:1	2274	39.03±15.07	73.72±12.96	21.17±9.19	22.31±7.29	286.37±45.7	204.69±36	127.38±48.64	76.72±15.81
7-C23:1	2281	96.33±39.3	127.1±33.84	42.51±18.56	67.99±22.26	1303.79±216.73	835.2±103.53	610.77±165.19	451.3±64.31
5-C23:1	2289	3.42±1.18	2.46±0.65	0.99±0.26	1.51±0.24	58.38±6.26	46.57±5.34	20.84±6.6	15.16±2.68
C23	2300	44.91±18.14	129.5±19.14	13.54±5.33	8.48±2.45	390.01±52	257.46±30.85	112.09±19.71	94.44±6.17
2-MeC23	2363			2.66±0.86	1.85±0.45			3.18±1.76	1.94±0.46
12-; 10-; 9-C24:1	2369	3.25±1.37	1.44±0.39						
3-MeC23	2373				0.99±0.33	1.14±0.55			
8-C24:1	2377	1.51±0.54	2.27±0.52		1.19±0.38	2.18±0.5	1.36±0.42	2.11±0.84	1.01±0.31
7-C24:1	2383		1.35±0.41		0.48±0.19	6.52±0.68	4.09±0.84	1.82±0.83	
6-C24:1	2386				0.54±0.22			1.13±0.61	
C24	2400	2.84±1.02	6.58±0.92	1.01±0.37	0.87±0.23	4.09±0.5	2.85±0.36	2.11±0.71	1.01±0.33
7,11-C25:2	2453	1±0.48		2.97±1.92	1.51±0.41			2.69±1.32	0.8±0.14
2-MeC24	2463	9.74±1.62	6.91±1.87	89.84±27.16	48.91±12.4	3.31±0.62		81.84±22.05	44.04±10.61
6,9-C25:2	2471	32.71±11.66	37.76±6.96			23.14±4.37	18.15±7.53	16.87±8.39	6.57±1.16
9-C25:1	2474	2.34±0.91		11.77±3.62	8.34±2.84	20.44±3.67	7.46±1.98	17.85±7.81	6.45±0.82
7-C25:1	2482	15.27±4.43	22.39±1.65	13.67±4.41	14.7±4.79	81.21±10.42	43.37±8.29	40.32±15.89	22.87±4.75

5-C25:1	2492					6.07±0.96	4.35±0.56	2.26±0.93	1.79±0.31
C25	2500	12.84±4.18	41.87±3.6	7.63±2.74	4.85±1.4	76.46±6.76	46.72±5.35	16.19±4.5	8.84±1.37
2-MeC25	2563			4.8±1.03	2.66±0.43			2.24±1.05	1±0.3
3-MeC25	2572			4.07±0.95	2.73±0.47				
9-; 8-C26:1	2578			0.6±0.24				1.28±0.42	
7-C26:1	2583			0.94±0.28	0.46±0.11				
5-C26:1	2588			2.06±0.59	0.85±0.3				
C26	2600	2.4±0.5	3.97±0.53	2.67±0.32	1.78±0.42			1.46±0.66	
X,Y-C27:2	2648			8.6±1.94	6.2±2.67				
7,11-C27:2	2656			21.65±3.34	13.82±1.87			5.76±3.7	1.36±0.3
2-MeC26	2663	32.01±4.27	30.59±6.54	251.4±37.12	123.3±11.32	6.22±1.18	1.71±0.34	144.81±51.49	71.44±16.24
11-C27:1	2673	21.07±2.41	6.97±1.57		28.23±4.75			21.74±12.55	9.43±3.4
9-C27:1	2676				15.32±1.34			24.53±11.99	6.77±1.5
7-C27:1	2684	20.6±2.4	9.26±1.93	58.33±8.2	28.43±3.84	2.49±0.54		25.2±10.84	9.77±1.79
X-C27:1	2694				0.66±0.15				
C27	2700	24.11±3.38	45.16±3.47	13.54±1.78	9.96±3.09	46.68±5.07	26.94±2.69	8.55±2	4.2±0.71
13-; 11-MeC27	2733	2.38±0.39		4.5±0.71	2.84±0.31			0.85±0.44	
7-MeC27	2742	6.24±0.83	1.62±0.38	5.43±0.76	3.65±0.85				
X,Y-C28:2	2747			6.44±1.19	5.55±2.03			1.19±0.75	
5-MeC27	2751	1.74±0.13		8.28±1.06	4.59±0.77				
2-MeC27	2763	4.36±0.32	3.07±0.44	6.25±0.68	3.83±0.36			1.54±0.59	
3-MeC27 + 14-; 13-; 12-; 11-; 10-; 9-C28:1	2773	5.54±0.51	1.81±0.39	10.57±1.05	10.35±1.73				
7-; 8-C28:1	2782	8.44±1.09	4.66±1.24	10.22±1.21	7.25±1.95				
6-C28:1	2790	5.48±0.43	2.43±0.78	15.84±2.26	8.94±1.12			1.86±1.12	1.14±0.71
C28	2800	6.45±0.54	7.59±0.85	4.98±0.41	4.25±1.27	1.48±0.35	0.97±0.13	1.19±0.59	0.97±0.46
X,Y-C29:2	2845							14.92±8.06	4.86±1.44
7,11-C29:2	2851	7.88±0.84	2.18±0.69	75.83±12.55	44.65±3.86			30.4±17.65	10.08±2.75
2-MeC28	2866	285.84±18.69	168.42±45.32	342.94±21.43	246.1±19.31	101.46±10.78	53.64±6.79	137.08±42.32	92.87±17.71
13-; 11-C29:1	2872	71.33±5.34	25±8.5						
9-C29:1	2877	59.15±5.87	21.53±5.59	46.89±3.52	30.07±4.63			14.69±8.52	
7-C29:1	2886	113.33±9.36	44.56±10.37	110.04±9.96	76.46±12.52	6.24±1.11	2.57±0.52	32.94±14.9	12.92±2.78
C29	2900	56.91±3.42	65.22±12.33	12.14±1.38	8.34±1.75	66.81±10.77	41.03±6.3		3.26±0.68
13-MeC29	2931	15±1.94	4.11±0.8	14.95±2.15	9.33±0.98	0.93±0.4	0.69±0.32	2.68±1.23	1.06±0.29
7-MeC29	2941	10.43±1.49	2.94±0.6	9.52±1.24	6.18±0.47				
W,X-C30:2	2950	2.87±0.7							
Y,Z-C30:2	2956	2.02±0.54							
2-MeC29	2964		2.23±0.27		1.5±0.53				
7,17-diMeC29	2973	14.75±1.95	4.31±0.92	8.33±0.87	6.76±1.24			2.61±1.36	0.69±0.27
8-; 10-; 12-C30:1	2982	9.93±1.29	3.83±1.07	8.78±1.08	7.75±2.03			1.77±0.95	0.48±0.23
C30	3300	7.32±2.09	2.75±0.53	1.39±0.26	1.84±0.43				
X,Y-C31:2	3048	24.83±4.04	4.22±1.6	13.13±3.33	5.77±0.98			2.63±1.35	0.82±0.24
7,11-C31:2	3054	34.49±4.01	8.25±1.82	10.61±1.62	6.37±0.9			2.74±1.49	0.72±0.23
2-MeC30	3064	127.09±10.75	54.3±7.05	35.06±6.7	21.2±4.7	9.94±2.38	3.73±0.62	7.05±2.76	4.69±1.51
6,9-C31:2	3069			5.97±1.78	4.28±1.39				
13-; 11-; 9-C31:1	3079	76.69±9.93	25.36±7.18	32.1±1.78	21.34±3.34			6.35±3.57	1.84±0.73
7-C31:1	3087	9.64±2.21	2.18±1.09	7.43±1.42	6.75±1				
C31	3100	5.96±0.59	5.79±1.4		0.79±0.45	1.38±0.4			
13-MeC31	3131	2.25±0.37	1.08±0.22	2.19±0.32	1.29±0.17				
W,X-C33:2	3257	20.66±2.29	2.79±0.86	5.31±1.49	2.49±0.39				
Y,Z-C33:2	3265	6.73±2.47	2.84±0.8	1.69±0.55	0.92±0.2				
**Total**		**1476.8**	**1207.8**	**1456.2**	**1022.1**	**3164.28825**	**2108.400944**	**1984.220696**	**1214.232882**

Supplementary Table S2: Number of tested *D. suzukii* pairs in mating trials. Discarded pairs: either the female or the male died during the behavioral experiment.

	winter ♀ X winter ♂	summer ♀ X winter ♂	winter ♀ X summer ♂	summer ♀ X summer ♂
Total tested pairs	19	17	16	15
Discarded pairs	4	2	1	0
Successful mating pairs	15	15	15	15
